# New Insights into Biochemical, Genotoxic, and Analytical Aspects of Low-Level Imidacloprid Exposure in Liver and Kidney Tissue of Adult Male Wistar Rats

**DOI:** 10.3390/toxics13100879

**Published:** 2025-10-15

**Authors:** Anja Katić, Vilena Kašuba, Nevenka Kopjar, Blanka Tariba Lovaković, Gordana Mendaš, Vedran Micek, Mirta Milić, Alica Pizent, Suzana Žunec, Ana Lucić Vrdoljak

**Affiliations:** 1 Division of Toxicology, Institute for Medical Research and Occupational Health, Ksaverska cesta 2, 10000 Zagreb, Croatia; vkasuba@imi.hr (V.K.); nkopjar@imi.hr (N.K.); btariba@imi.hr (B.T.L.); mmilic@imi.hr (M.M.); suzana@imi.hr (S.Ž.); alucic@imi.hr (A.L.V.); 2 Division of Environmental Hygiene, Institute for Medical Research and Occupational Health, Ksaverska cesta 2, 10000 Zagreb, Croatia; gmendas@imi.hr; 3 Animal Breeding Unit, Institute for Medical Research and Occupational Health, Ksaverska cesta 2, 10000 Zagreb, Croatia; vmicek@imi.hr; 4 Division of Occupational and Environmental Health, Institute for Medical Research and Occupational Health, Ksaverska cesta 2, 10000 Zagreb, Croatia; apizent@imi.hr

**Keywords:** neonicotinoids, low-level exposure, cholinesterase, oxidative damage, genotoxicity, HPLC-UV DAD analysis

## Abstract

Besides its neurotoxic action and selective toxicity on insecticidal nicotinic acetylcholine receptors, recent studies have shown that imidacloprid may cause other adverse effects in mammals. In the present study, cholinesterase activity, oxidative stress response, genotoxicity in the liver and kidney, and imidacloprid levels in the urine, liver, and kidney of male Wistar rats orally administered with 0.06, 0.8, and 2.25 mg imidacloprid/kg bw/day for 28 days were evaluated. Imidacloprid urine mass concentrations in treated rats increased dose-dependently. Exposure to 0.8 mg imidacloprid/kg bw/per day significantly decreased cholinesterase activities in the liver and kidney. Reactive oxygen species levels decreased significantly in the liver at the same dose. Lipid peroxidation was significantly reduced in the liver at two higher doses. No significant changes in glutathione levels or the activities of superoxide dismutase and catalase were observed. A significant decrease in the activity of glutathione peroxidase was detected in the liver at the highest dose. DNA damage was low in both liver and kidney. Exposure to imidacloprid at studied experimental conditions did not cause a significant oxidative stress response and resulted in low genotoxic effects in the liver and kidney of rats, indicating that these organs are less susceptible to adverse imidacloprid effects at such low doses.

## 1. Introduction

Imidacloprid is a systemic insecticide belonging to the class of neonicotinoid pesticides, introduced to the market in 1991 [1]. It is used mostly for seed/soil treatments to control biting, sucking, and some chewing insects on a variety of crops, in households, and in veterinary medicine on pets [1,2]. Imidacloprid affects the central nervous system by acting as an agonist of nicotinic acetylcholine receptors (nAChRs). By irreversibly binding and blocking nAChRs, imidacloprid prevents acetylcholine from transmitting neural impulses and causes overstimulation, paralysis, and death of the pests. The selective action of imidacloprid due to structural differences in nAChRs between insects and mammals, and its lower binding affinity to mammalian neuron receptors and lower toxicity to mammals than to the insects, contributes to its auspicious toxicological profile [3,4].

Human exposure to imidacloprid occurs mostly from food and drinking water as a result of its direct application to growing crops and oyster beds and its potential to reach sources of drinking water. Also, exposure through residential use and occupational settings presents exposure pathways for risk assessment [5]. Like other neonicotinoids, imidacloprid is characterized by photostability, resistance to hydrolysis, and high water solubility. Due to agricultural runoff during rainfall or irrigation, from treated crop fields it may enter the aquatic ecosystem. Also, through leaching, it may enter the groundwater and surface water, thus ending up in drinking water [6]. Imidacloprid’s half-life in the soil is up to 190 days, and in water, greater than 31 days [7]. In addition, the metabolism of imidacloprid in the environment is very low [8]. Due to all these characteristics, imidacloprid is considered a persistent environmental pollutant. It fulfills very persistent (vP) and toxic criterion; however, it does not fulfill bioaccumulative (B) and very bioaccumulative (vB) criterion based on the fish and earthworm toxicity assessment. Thus, it is not classified by the European Chemicals Agency (ECHA) either as persistent, bioaccumulative and toxic (PBT) or very persistent and very bioaccumulative (vPvB) [9]. Based on this classification, production of imidacloprid in the EU is approved until 2025, and application for renewal of the authorization for further production is under review [10].

Although neonicotinoids were initially considered to be low risk for non-target organisms, imidacloprid has been specified in numerous studies as highly toxic for honeybees and bumblebees, and has also been labeled responsible for the decline in their populations [11,12,13,14,15,16]. These significant pollinators, like other insects, are target organisms for the action of imidacloprid, related to binding to the nAChRs. Thus, it represents a significant risk for biodiversity and agriculture, i.e., fruit formation in all plants pollinated by insects. Unlike the ECHA, the European Food Safety Authority (EFSA) banned the use of imidacloprid for outdoor uses in 2018, except for permanent greenhouses, and there is no application for renewal of approval for its usage [17].

Rapid and high absorption rates of imidacloprid have been shown in the gastrointestinal tract of rats (more than 92%), as well as in human intestinal CaCo-2 cells [18,19]. It is distributed rapidly and broadly within 1 h after oral administration. Imidacloprid is mostly metabolized in the liver, forming three main metabolites: 5-hydroxy and olefin derivatives excreted both in feces and urine; and 6-chloronicotinic acid (CNA) and its glycine conjugate excreted exclusively through urine in rodents [18,20]. In humans, 4 and 5 hydroxylated imidacloprid, olefin, and 6-CNA-glycine derivatives were detected in the urine after oral exposure to the corresponding acceptable daily intake (ADI) dose [21]. In addition, the rapid and high excretion rate has been established in rats with complete excretion of 96% imidacloprid within 48 h and more than 90% ingested imidacloprid within 24 h, primarily via the urine (75%) and less through feces (25%) [18,20].

Although imidacloprid has been considered safer than other insecticides due to its selective action and favorable toxicological profile, studies relevant for risk assessment in humans have shown that it may induce adverse health effects in mammals. While acute imidacloprid exposure neurotoxicity is the most delicate endpoint, in long-term exposure, other toxicities like immunotoxicity, developmental toxicity, as well as developmental neurotoxicity, teratogenicity, and reproductive toxicity have been observed [5,22,23]. Imidacloprid may also act as an endocrine-disruptive chemical [23,24,25,26]. There is no evidence that imidacloprid has carcinogenic potential; thus, it has been classified as a Group E chemical showing non-carcinogenicity for humans [27]. Recent in vitro and in vivo findings have shown imidacloprid genotoxic potential as a result of DNA damage, chromosomal alteration, and gene mutation [28,29,30,31,32,33,34,35,36,37]. Different organ toxicities, including liver and kidney, have been observed after imidacloprid administration in experimental animals. Increases in reactive oxygen species (ROS) production and oxidative stress have been noticed as proposed mechanisms of cellular and organ damage [37,38,39,40,41,42,43].

Due to the hazards of consuming food that contains residues of imidacloprid, it is necessary to elucidate its toxicity in non-target organisms to minimize health risk. It is particularly important to investigate exposure to doses relevant to real human exposure, especially since there is a lack of such research. In the present study, we aimed to evaluate toxic and genotoxic effects of low doses of imidacloprid encountered in a realistic exposure scenario, comparable to the reference values for imidacloprid stipulated in the current EU legislation, in male Wistar albino rats. After repeated 28-day oral exposure, we assessed acetylcholinesterase (AChE, EC 3.1.1.7) and butyrylcholinesterase (BChE, EC 3.1.1.8) activities and the parameters of oxidative stress, which comprise ROS and glutathione (GSH) levels, lipid peroxidation (LPO), and the activities of catalase (CAT), superoxide dismutase (SOD), and glutathione peroxidase (GSH-Px), as biomarkers of toxicity. Genotoxicity of imidacloprid was evaluated by determining the level of primary DNA damage in liver and kidney cells. Complementary to the biochemical and genotoxic endpoints, the levels of imidacloprid in the liver and kidney tissue, as well as in urine, were assessed.

## 2. Materials and Methods

### 2.1. Chemicals and Reagents

Imidacloprid (CAS number 138261-41-3), as the analytical standard PESTANAL^®^ (MilliporeSigma, Buchs, Switzerland), and the positive control substance recommended for in vivo comet assay ethyl methanesulphonate (EMS) (CAS Number: 62-50-0) were purchased from Sigma-Aldrich Laborchemikalien GmbH, Darmstadt, Germany.

All other chemicals and reagents used for biochemical analysis and in the alkaline comet assay were of analytical grade and purchased from Sigma Chemical Co., Ltd., St. Louis, MO, USA, unless specified otherwise.

### 2.2. Animals and Experimental Design

Adult, three-month-old male Wistar HsdBrlHan rats (*Rattus norvegicus* sp.) were obtained from an inbred colony at the Institute for Medical Research and Occupational Health (IMROH, Zagreb, Croatia). Animals were randomly assigned to experimental groups, five animals per group, with minimal weight variation. They were kept in standard clear polycarbonate cages (Ehret, Tulln, Austria) with appropriate enrichment, under pathogen-free conditions, in steady-state microenvironmental conditions (12 h light/dark cycle, room temperature 20–22 °C, and humidity 40–60%). Animals had ad libitum access to standard Good Laboratory Practice (GLP)-certified food (complete feed for mice and rats 4RF21, Mucedola, Settimo Milanese, Italy) and tap water. This study was carried out in accordance with Directive 2010/63/EU of the European Parliament and of the Council on the protection of animals used for scientific purposes [44], and in compliance with the ARRIVE guidelines and all international standards and national legislation for the protection of animal welfare. It was approved by the Ethics Committee of IMROH, Zagreb, Croatia, and the Croatian Ministry of Agriculture (Reg No. 100-21/14-5, Class 01-18/14-02-2/6 of 11 June 2014).

All groups of animals were exposed orally by gavage with 1 mL of treatment solution daily for 28 consecutive days (except the positive control group) and were handled in the same manner. Three groups were treated with imidacloprid solutions that were prepared freshly before application by dissolving tested chemical in 0.03% ethanol (EtOH) and further diluted in distilled water. The selected doses of imidacloprid were based on toxicological reference values proposed by EFSA [45] and EU Pesticides Database [46], and represent real environmental exposures. We tested the effects of imidacloprid at the acceptable daily intake (ADI) level of 0.06 mg/kg bw/day, at 10× the acceptable operator exposure level (AOEL) of 0.8 mg/kg bw/day, and at the acute oral 1/200 LD_50_ level for rats of 2.25 mg/kg bw/day. Appropriate negative, positive, and solvent controls were investigated in parallel. The negative control group received water, the solvent control group received 0.03% EtOH, while the positive control group received a well-established genotoxicant recommended for in vivo comet assay in rodents, ethyl methanesulphonate (EMS), at a dose of 300 mg/kg bw/day [47], which was administered during the last three days of the experiment.

During the whole experiment, rats were weighed weekly and the doses of imidacloprid adjusted accordingly. Monitoring of animals and evaluation of survival and clinical signs of intoxication were carried out on a daily basis.

At the end of the 28-day exposure period, all animals were sacrificed 24 h after the final administration by exsanguination using an anesthetic cocktail (Narketan, Vetoquinol UK Ltd., Towcester, UK, 80 mg/kg bw; Xylapan, Vetoquinol UK Ltd., Towcester, UK, 12 mg/kg bw, i.p.). Liver and kidney tissues were harvested and dissected, rinsed with cold PBS, and divided into two portions. Tissue samples for biochemical and HPLC analysis were immediately frozen and stored at −20 °C until further processing, and samples used in the alkaline comet assay were prepared as described in the text below.

The 24 h urine samples were collected from the metabolism cage on the 1st, 14th, and 28th days of the treatment for analysis of imidacloprid and its metabolites in urine, and stored at −20 °C.

### 2.3. Biochemical Analysis

To determine cholinesterase activity, liver and kidney tissues were homogenized in 50 mmol/L potassium phosphate buffer containing 1 mmol/L EDTA (pH 7.4) at a 20% (*w*/*v*) ratio. For the measurement of oxidative stress markers, liver and kidney tissue samples were homogenized in 50 mmol/L potassium phosphate buffer containing 1 mmol/L EDTA (pH 7.8) at a 10% (*w*/*v*) ratio. The homogenates were centrifuged at 20,000× *g* for 30 min at 4 °C (Mikro 22R centrifuge, Hettich, Tuttlingen, Germany), and the resulting supernatants were used for analysis of antioxidant enzyme activities. All measurements were performed in triplicate.

#### 2.3.1. Cholinesterase Activity Assay

Liver and kidney tissue samples were analyzed for total cholinesterase (ChE), AChE, and BChE activities using Ellman’s spectrophotometric method, according to the previously described protocol [48,49]. All of the measurements were performed on a Cecil 9000 Spectrophotometer (Cecil Instruments Limited, Cambridge, UK). Enzyme activity was expressed as IU/g protein.

#### 2.3.2. Determination of Oxidative Stress Parameters

The levels of ROS and GSH were measured in 1% (*w*/*v*) liver and kidney homogenates prepared by dilution of 10% homogenates with ice-cold PBS (pH 7.4), as described in a previously published paper [37]. Fluorescence intensity was measured using a Victor3TM (PerkinElmer, Inc., Waltham, MA, USA) plate reader, and data were expressed in arbitrary fluorescence units (AU).

The concentration of thiobarbituric reactive substances (TBARS), as a measure of lipid peroxidation, was determined in 10% (*w*/*v*) tissue homogenates using a previously described protocol [49]. Absorbance was measured at 532 nm using a UV Probe Spectrophotometer (Shimadzu, Kyoto, Japan). TBARS concentrations were calculated using a standard curve of increasing 1,1,3,3-tetramethoxypropane concentrations, and expressed as μmol/L.

Activities of GSH-Px, SOD, and CAT in liver and kidney homogenates were determined spectrophotometrically. Procedures for sample preparation and measurements are described in previous studies [37,49,50]. Results are expressed as IU/g protein.

Protein concentration was measured according to the method of Bradford using bovine serum albumin as standard, as stated previously [49].

### 2.4. Determination of Primary DNA Damage with Alkaline Comet Assay

The assay was performed following the protocol by Singh et al. [51] with minor modifications. Tissues were prepared within one hour following animal sacrifice. Liver and kidney tissue samples were isolated, cleaned, washed in cold PBS buffer, drained, put into cold mincing buffer [75 mM NaCl (Kemika, Zagreb, Croatia), 24 mM Na_2_EDTA, pH 7.5], and carefully minced with a pair of fine scissors to release single cells. The obtained cellular suspensions were left for a few seconds on ice, and the supernatants were immediately used to prepare agarose microgels for the alkaline comet assay. Two coded gel replicates were prepared per tissue for each rat.

Agarose microgels were prepared as described previously [37,49,50]. After solidification, the gels were submerged overnight at 4 °C in a freshly prepared cold lysing solution [100 mmol/L Na_2_EDTA, 2.5 mol/L NaCl, 1% Na-laurylsarcosinate, 10 mmol/L Tris-HCl, 10% dimethyl sulfoxide (Kemika, Zagreb, Croatia), and 1% Triton X-100, pH = 10]. Slides were quickly washed with distilled water and left in a vertical Coplin jar for 10 min at +4 °C in chilled, freshly made alkaline electrophoresis buffer [1 mmol/L Na_2_EDTA and 300 mmol/L NaOH (Kemika, Zagreb, Croatia), pH > 13] and kept in the dark. Then, they were placed into a horizontal electrophoresis unit (Horizon 11.14, Whatman, Maidstone, UK). Electrophoresis was conducted at 1 V/cm for 10 min at +4 °C with a constant voltage of approximately 300 mA. After electrophoresis, slides were washed three times at five-minute intervals with neutralization buffer (0.4 M Tris-HCl, pH 7.5), dehydrated with 70% and 96% ethanol, air dried, and stored until scored at room temperature. They were stained with ethidium bromide (20 μg/mL) and analyzed under a fluorescent microscope (Olympus BX51, Olympus, Hachioji, Tokyo, Japan) equipped with appropriate filters, under 200× magnification, and using an image analysis system Comet Assay IV^TM^ (Instem-Perceptive Instruments Ltd., Suffolk, Halstead, UK). One well-trained scorer performed all of the comet measurements. Per tissue sample, per point, and per animal, 300 cells (two gels, with 150 cells measured per gel) were scored. Measurement criteria included the selection of random fields on the microgel, avoiding areas around air bubbles or at the edges [52]. All comets were captured at a constant depth of the gel.

The tail intensity (i.e., DNA% in tail) and tail length (presented in micrometres) were chosen as descriptors of DNA damage. Comets with >80% DNA in the tail region, or comets with small or non-existing heads and large, diffuse tails were classified as “hedgehog” cells and were excluded from the analysis. Since two cell types are present in liver samples, medium-sized cells (parenchymal cells or hepatocytes, between 30 and 40 μm head length) and small-sized cells (non-parenchymal cells, <30 μm of head length), these were recorded separately [53].

### 2.5. HPLC-UV DAD Analysis

Imidacloprid, 6-chloronicotinic acid, and imidacloprid urea (all 99% purity) were purchased as analytical standards from Dr. Ehrenstorfer GmbH (Augsburg, Germany). Acetonitrile, methanol, ethyl acetate, and acetone (ultra-gradient HPLC grade) were obtained from J.T. Baker (Deventer, The Netherlands). LC-grade water was prepared using a Milli-Q water purification system (Millipore, Bedford, MA, USA). All other chemicals used were of pro-analysis purity from Kemika (Zagreb, Croatia). For solid-phase extraction (SPE) of compounds from urine samples, Bakerbond SDB/C18 cartridges (6 mL, 100 mg; J.T. Baker, Deventer, The Netherlands) were utilized.

Model urine samples were prepared at three concentration levels within the calibration range (0.04–1.20 µg/mL; five replicates per level) by spiking untreated rat urine with a standard mixture of imidacloprid, 6-chloronicotinic acid, and imidacloprid urea. Blank samples were processed in parallel. Extraction recoveries in this range were 91–95% with an RSD < 7%.

High-performance liquid chromatography with a UV diode-array detector (HPLC-DAD) was used to quantify imidacloprid and its metabolites in urine from treated rats. The analysis was performed using a Varian ProStar HPLC system (Varian, Palo Alto, CA, USA) with a Gemini C18 column (250 mm × 4 mm I.D., 5 µm particle size, Phenomenex, Torrance, CA, USA). The mobile phase consisted of acetonitrile and 0.5% formic acid in water, with a gradient elution from 10% to 70% acetonitrile. The flow rate was 1 mL/min, and the injection volume was 100 µL. Compounds were identified by retention time and UV spectra compared with standards. Calibration curves (five concentration levels, triplicate) showed linearity in the range 0.04–1.20 µg/mL. The detection limit for imidacloprid was 20 ng/mL.

The amount of imidacloprid in urine was calculated as the total mass in 24 h samples. While urine is often treated as a relatively homogeneous biological matrix for quantitative analyses, we acknowledge that it can exhibit matrix variability; for this reason, all urine samples from treated animals were analyzed in duplicate (two parallel preparations).

### 2.6. Statistical Analysis

Statistical data analysis was performed by the Dell™ Statistica™ licensed statistical software package Version 13.5.0.17 (TIBCO Software Inc., Palo Alto, CA, USA).

Firstly, the normality of (Gaussian) distribution was tested with the Shapiro–Wilk test.

Results of cholinesterase activities and biochemical markers of oxidative stress were not normally distributed, and were analyzed by the non-parametric Kruskal–Wallis test and multiple comparisons test with Bonferroni’s correction.

The non-parametric Mann–Whitney U test was applied for between-group comparisons of the alkaline comet assay since results were not normally distributed even after logarithmic transformation.

Imidacloprid concentrations in urine were normally distributed, and multiple comparisons between treatment groups were performed using one-way analysis of variance (ANOVA) following post hoc comparisons with Bonferroni’s correction.

Statistical significance was set at *p* values < 0.05.

## 3. Results

Treatment with imidacloprid for 28 days did not cause any signs of systemic toxicity or death in male adult Wistar rats. In the course of this study, rats exposed to imidacloprid did not show marked behavioural changes, nor signs of incoordination, ataxia, tremors, or any signs typical of cholinergic overstimulation compared to control rats. Gross necropsy did not reveal any treatment-related findings.

### 3.1. Cholinesterase Activities

**Figure 1:**
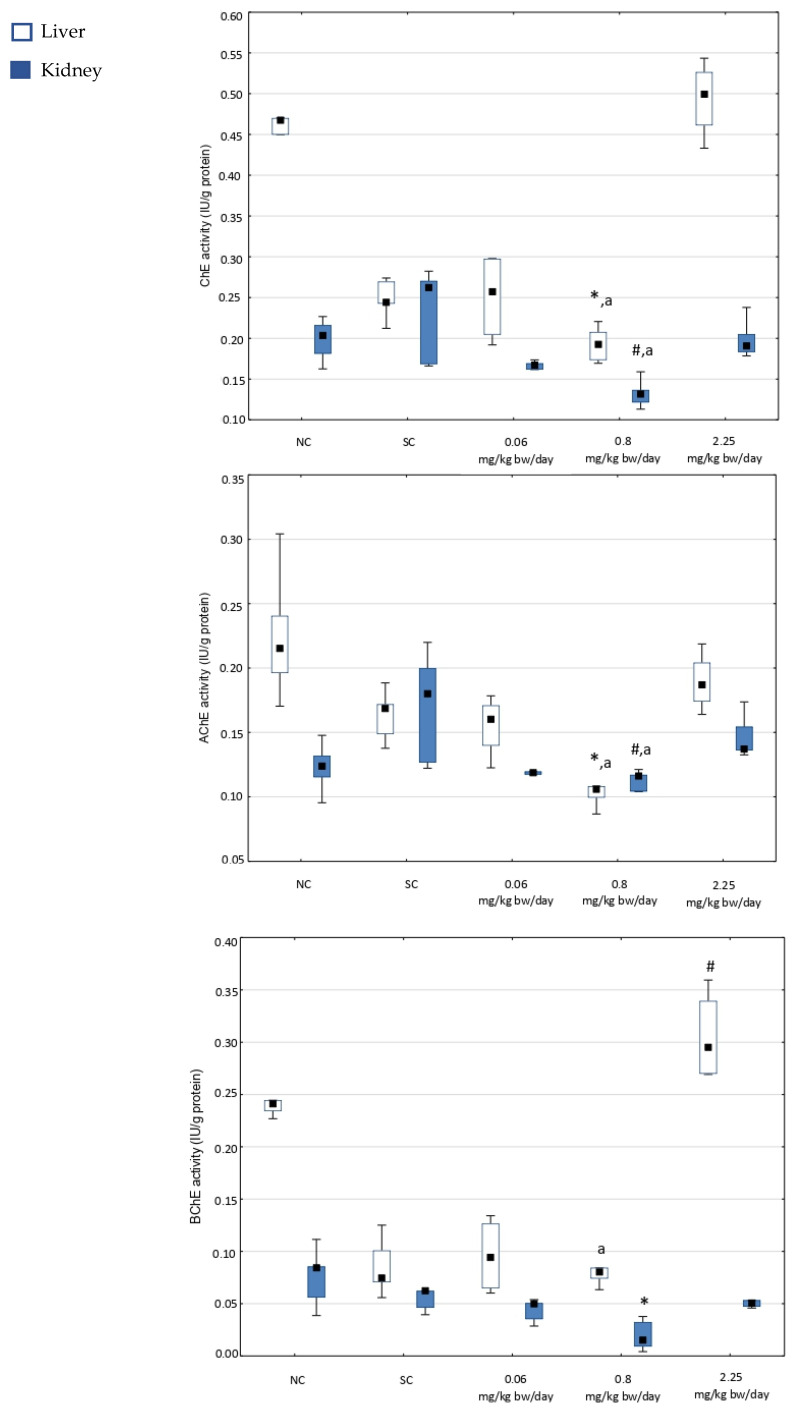
Total cholinesterase (ChE), acetylcholinesterase (AChE), and butyrylcholinesterase (BChE) activities in the liver and kidney tissues of adult male Wistar rats treated orally for 28 consecutive days with imidacloprid applied at three doses and in the corresponding controls (*N* = 5 rats per group). NC—negative control, SC—solvent control. The results are shown as median (square), 25th and 75th percentiles (box), and min–max range (whisker). Statistically significant values (set at *p* < 0.05; Kruskal–Wallis test) are as follows: *—vs. negative control; ^#^—vs. solvent control; ^a^—vs. 2.25 mg/kg bw/day.

Figure 1 presents the effects of imidacloprid on the catalytic activity of total ChE, AChE, and BChE in the liver and kidney tissues of male Wistar rats.

In the liver, overall statistical significance was observed for ChE activity (χ^2^ = 18.99; df = 4; *p* = 0.0008), AChE activity (χ^2^ = 12.18; df = 4; *p* = 0.0161), and BChE activity (χ^2^ = 16.79; df = 4; *p* = 0.0021). A dose of 0.8 mg imidacloprid/kg bw/day significantly reduced the activities of ChE and AChE compared to the negative control, as well as the activities of ChE, AChE, and BChE compared to the highest imidacloprid dose of 2.25 mg/kg bw/day. In contrast, the activities of ChE and AChE in the liver of rats treated with the highest dose of imidacloprid were similar to those in the negative control group, while the activity of BChE increased significantly compared to solvent control rats.

In the kidney tissue, overall statistical significance was observed for ChE activity (χ^2^ = 16.99; df = 4; *p* = 0.0019) and AChE activity (χ^2^ = 13.78; df = 4; *p* = 0.0080). There were no significant differences in ChE, AChE, and BChE activities in kidney tissue at the lowest and highest doses of imidacloprid. However, administration of the dose of 0.8 mg imidacloprid/kg bw/day significantly reduced ChE and AChE activities compared to both the solvent control and the highest dose of imidacloprid (2.25 mg/kg bw/day), and also reduced BChE activity compared to the negative control.

Overall, in both the liver and kidney, the activities of all cholinesterases were mostly inhibited by imidacloprid treatment at the dose of 0.8 mg/kg bw/day, while the highest dose of imidacloprid affected cholinesterase activities the least.

### 3.2. Oxidative Stress Parameters

**Figure 2:**
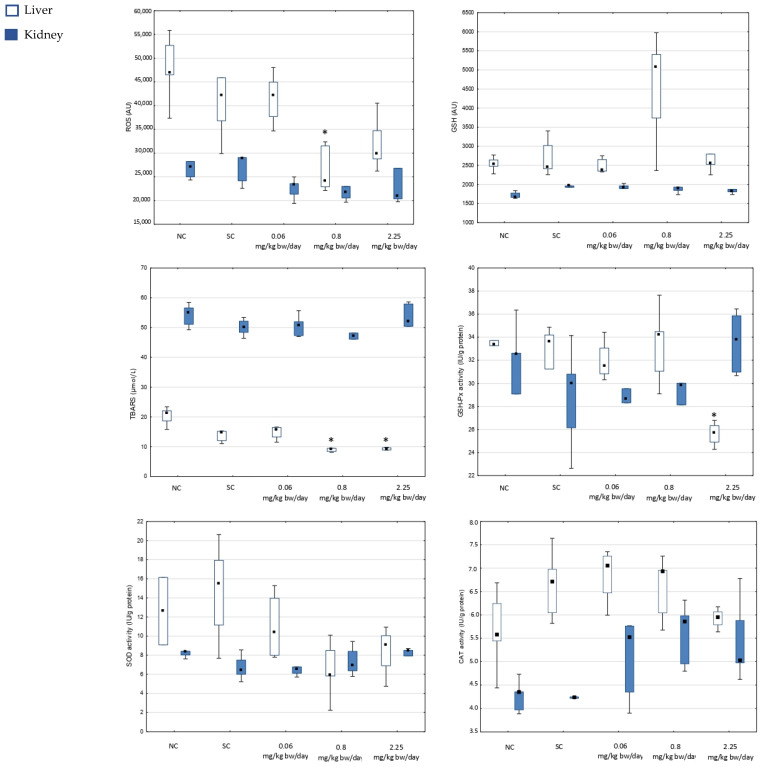
Results regarding the biochemical markers of oxidative stress measured in the liver and kidney of adult male Wistar rats treated orally for 28 days with imidacloprid applied at three doses, and in the corresponding controls (*N* = 5 rats per group). ROS—reactive oxygen species; GSH—glutathione; TBARS—thiobarbituric reactive substances; GSH-Px—glutathione peroxidase; SOD—superoxide dismutase; CAT—catalase. NC—negative control, SC—solvent control. The results are shown as median (square), 25th and 75th percentiles (box), and min–max range (whisker). Statistically significant values (set at *p* < 0.05; Kruskal–Wallis test) are as follows: *—vs. negative control.

The measured levels of ROS, GSH, LPO expressed as TBARS concentration, and the activities of antioxidant enzymes GSH-Px, SOD, and CAT in the liver and kidney tissues of male Wistar rats are illustrated in Figure 2.

Oral exposure to imidacloprid decreased ROS levels in the liver tissue, with a statistically significant reduction only at the dose of 0.8 mg imidacloprid/kg bw/day compared to negative control rats. Overall, a statistically significant difference was observed in the ROS levels (χ^2^ = 10.58; df = 4; *p* = 0.0318). The ROS levels in the kidney tissue slightly decreased after exposure to all three doses of imidacloprid in comparison to the respective control groups.

The dose of 0.8 mg/kg bw imidacloprid/per day increased GSH levels in the liver tissue, although this change was not statistically significant. GSH levels in the liver tissue following exposure to the lowest and to the highest dose, as well as in the kidney tissue after exposure to all three doses of imidacloprid, were comparable to those in the negative control group.

Imidacloprid treatment decreased TBARS concentrations in the liver significantly at doses of 0.8 and 2.25 mg/kg bw/per day compared to negative control rats. Overall, a statistically significant difference was observed in the TBARS concentrations (χ^2^ = 15.19; df = 4; *p* = 0.0043). The concentration of TBARS in kidney changed slightly.

Significantly decreased activity of GSH-Px was observed in the liver tissue after exposure to the highest dose of imidacloprid (2.25 mg/kg bw/per day) relative to the negative controls. GSH-Px activity in the kidney tissue of rats exposed to imidacloprid was slightly disturbed.

In the liver and kidney tissues, exposure to all three doses of imidacloprid disturbed SOD activity, but significant changes were not observed. SOD activity was decreased in the liver after exposure to all three doses of imidacloprid, and in the kidney following exposure to two lower doses of imidacloprid.

Imidacloprid exposure did not significantly affect CAT activity in either liver or kidney tissue. The levels of CAT activity at all three doses of imidacloprid were higher than in respective controls in the liver and kidney, with a dose-dependent decrease in the liver.

### 3.3. The Alkaline Comet Assay

The results of the alkaline comet assay, expressed as tail intensity (DNA%) and tail length (µm) measured in small-sized (non-parenchymal) and medium-sized (parenchymal) liver cells, are presented in Table 1.

Regarding the results obtained for small-sized liver cells, the negative control showed very low background values for both comet assay descriptors. Those values were slightly higher in the solvent control group. As expected, exposure to the genotoxic chemical EMS led to a significant increase in both comet assay descriptors. Imidacloprid exposure slightly affected the values of both descriptors, and their statistical significance is indicated in Table 1. However, if we consider the obtained numerical values of group means and medians, the tested doses of imidacloprid caused a low level of genotoxicity. Nevertheless, it is clear that the treatments result in different degrees of damage in individual cells, as indicated by the maximum values visible in the measured ranges. If we look at the data obtained for medium-sized liver cells, a very similar trend is noticeable. When focusing only on the three imidacloprid-exposed groups, without considering the controls, the fact is that, in mathematical terms, the highest measured values—both in small-sized liver cells and medium-sized liver cells—were recorded following exposure to a dose of 0.06 mg imidacloprid/kg bw/per day.

Table 2 reports the results of the alkaline comet assay expressed as tail intensity (DNA%) and tail length (µm) measured in kidney cells. The negative control showed very low background values for both comet assay descriptors. Those values were slightly higher in the solvent control group. As expected, exposure to the genotoxic chemical EMS led to an increase in both comet assay descriptors. Exposure to imidacloprid slightly changed the values of both descriptors, and their statistical significance is indicated in Table 2. Similar to the case of both types of studied liver cells, if we consider the obtained numerical values of group means and medians, the tested doses of imidacloprid caused a low level of genotoxicity in kidney cells. Here too, the treatments result in different degrees of damage in individual cells, as indicated by the maximum values visible in the measured ranges. When focusing only on the three imidacloprid-exposed groups, without considering the negative/solvent controls, the fact is that, in mathematical terms, the highest median values in kidney cells were recorded after treatment with the lowest imidacloprid dose (0.06 mg/kg bw/day).

### 3.4. Analysis of Imidacloprid and Its Metabolite in Urine

**Figure 3:**
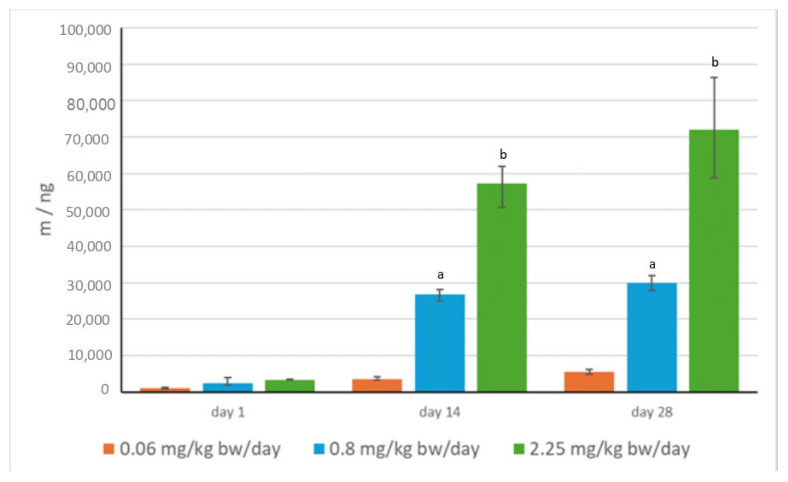
Urinary excretion of imidacloprid in 24 h urine samples of male Wistar rats treated orally for 28 consecutive days with three doses of imidacloprid, collected on days 1, 14, and 28 of treatment (*N* = 5 rats per group). The results are presented as means and ranges (error bars denote minimum and maximum values). Repeated-measures ANOVA with Bonferroni-corrected post hoc tests (*p* < 0.05): ^a^—vs. day 1 at dose 0.8 mg/kg bw/day; ^b^—vs. day 1 at dose 2.25 mg/kg bw/day.

Results of HPLC-UV DAD analysis of 24 h urine from imidacloprid-treated rats are presented in Figure 3.

Imidacloprid, as a parent compound, was confirmed in all 24 h urine samples. The mass concentrations of analyzed metabolites were below the detection limit of 20 ng/mL. Imidacloprid urinary levels were dose-dependent and increased with the duration of exposure. The highest concentrations were measured in the urine of rats treated with the highest dose of imidacloprid of 2.25 mg/kg bw/per day, collected at the end of the experiment.

The repeated-measures ANOVA indicated a clear effect of time on urinary excretion of imidacloprid, which was confirmed statistically (F(2,24) = 66.18, *p* < 0.001), as well as a significant interaction between dose and time (F(4,24) = 18.79, *p* < 0.001), indicating that the pattern of excretion varied between treatment groups. Although the main effect of dose alone was not statistically significant (F(2,12) = 2.43, *p* = 0.13), post hoc comparisons (Bonferroni-corrected) confirmed a significant increase in excreted mass between sampling days in the medium- and high-dose groups (*p* < 0.05), while the low-dose group showed no significant changes over time.

Neither imidacloprid nor its metabolites were detected at quantifiable levels in the liver and kidney tissues of imidacloprid-exposed rats. Chromatographic analysis of these samples was limited by matrix interferences and compound concentrations, which were below the limit of quantification.

## 4. Discussion

Since their introduction to the market, neonicotinoids have become the fastest-growing class of chemical insecticides with an efficient mode of action and safer use than other insecticides, and due to this, they have replaced some other chemical classes of insecticides. In the existing literature, there are conflicting results about their toxicity, which relate to exposure to high doses, among other factors, and do not reflect real-life exposure levels. Also, since they are considered safe for humans at low concentrations, there is limited research regarding long-term health effects. Exposure to neonicotinoids can occur through different routes. Due to systemic accumulation of neonicotinoids and chronic exposure, food presents a very important route of human exposure. The adverse outcome pathways (AOPs) for the toxicity of neonicotinoids in mammals have not been established yet, so there is a need to investigate potential adverse health effects related to chronic environmental exposure using integrating studies. Thus, in this study, we tried to clarify whether a repeated 28-day oral exposure to low and real human doses of imidacloprid causes signs of toxicity in adult male Wistar rats by assessing different biomarkers in liver and kidney tissue.

The results of detectable imidacloprid levels in the urine of treated rats, measured with the HPLC-UV DAD method, have shown that at all sampling points detected, imidacloprid mass concentrations increased dose-dependently. Although imidacloprid has a quick excretion rate, long-term exposure in our investigation enhanced the detected mass levels in the urine with each subsequent sampling point, which could be explained by its accumulation in kidney tissue. The study by Nimako et al. [54] has shown that the kidney accumulates imidacloprid significantly less than testis, blood, brain, and lung tissue but more than adipose tissue and the pancreas, while imidacloprid was not detectable in the liver of male mice after chronic exposure of 24 weeks to a low dose of 0.6 mg imidacloprid/kg bw/day through diet. In addition, the same research has shown that testis, brain, and kidney tissue are the most susceptible to imidacloprid and its metabolite accumulation. In our study, we did not determine imidacloprid in the liver tissue of treated rats. Investigation in humans has shown only a small percentage of imidacloprid in urine of about 12.7% after oral ingestion, explained by probable transformation into metabolites that accumulate in different body tissues and organs [55]. Results of the pharmacokinetic study have shown that after a single oral dose of 20 mg imidacloprid/kg bw in female rats, imidacloprid was widely distributed and metabolized in different organs and excreted in urine, where the highest maximum concentration was observed [56].

In this study, we evaluated the effects of imidacloprid exposure on the activity of cholinesterase enzymes since they may serve as biomarkers of insecticide toxicity due to their important role in nerve transmissions and homeostasis [57]. Although imidacloprid is not a ChE inhibitor [58], it acts on nAChRs that are located in the central and peripheral nervous systems, but are also found in non-neuronal tissues [59]. Both AChE and BChE are structurally and functionally related cholinesterases responsible for hydrolyzing acetylcholine (ACh) [60]. If those enzymes are inhibited, ACh is not degraded quickly. This causes persistent stimulation of nAChRs and prolonged depolarization, and over time, can lead to receptor desensitization, excitotoxicity, and cell dysfunction. Repeated 28-day exposure to a dose of 0.8 mg imidacloprid/kg bw/per day in our study significantly reduced ChE and AChE activities in the liver tissue and BChE in the kidney tissue. Because compensatory upregulation or altered metabolism at high doses can restore measured activity, the partial recovery of AChE/BChE at the highest tested dose of 2.25 mg imidacloprid/kg bw/day is biologically plausible [61]. Previous studies have shown that exposure to imidacloprid at doses of 170 mg/kg i.p. for 12 and 24 h in rats of both sexes caused sex-, tissue-, and duration-specific effects on total cholinesterase activity in kidney and liver tissue [42].

Imidacloprid can affect multiple organs, including the liver and kidneys, due to its systemic properties. Liver is responsible for the metabolism and elimination of toxicants from the body, and it is the first major organ exposed to ingested toxins [62]. Considering the role of the liver in imidacloprid toxicity, investigations of histological and biochemical parameters are important to clarify the health risk from chronic exposure. Imidacloprid can damage the liver morphology and function in different ways. Previous research on rodents of both sexes has shown that long-term imidacloprid exposure that lasted from 21 to 90 days caused histopathological changes in the liver that correlated with disruption of liver enzymes [40,41,63,64,65,66,67,68,69,70,71,72,73,74]. Kidney is the major detoxification organ and a target organ for most xenobiotics as proximal tubular cells are able to accumulate slightly acidic and basic compounds, amino acid conjugates, and quaternary compounds at toxic levels [75]. Also, renal proximal tubules are targets for toxicity partly due to the expression of transporters that mediate the elimination and reabsorption of xenobiotics [76]. Histopathological changes were revealed in the kidney tissue of rodents of both sexes exposed to imidacloprid from 15 to 90 days [63,65,67,69,71,77,78].

Oxidative stress may also have an important role in different toxicities of imidacloprid, which has been shown in several studies on non-target organisms during the last decade. It is known that mitochondria are an important target of neonicotinoids’ action. Since they play an important role, among other things, in redox homeostasis, damage may be fatal to a cell and, consequently, for the whole organism [79]. Mitochondria are also the main source of ROS, whose overproduction may contribute to mitochondrial oxidative damage [80]. Producing oxygen free radicals due to exposure to neonicotinoids can cause tissue damage by triggering some oxidative mechanisms as well as lipid peroxidation. Malondialdehyde (MDA) and TBARS, as the measure of LPO, are one of the most important markers of oxidative stress. Many studies have shown that neonicotinoids can increase LPO [81]. For instance, increased MDA levels have been observed in the kidney and liver tissue of male and female mice exposed orally to a dose of 15 mg/kg imidacloprid for 21 days [65]. Exposure to imidacloprid at a dose of 64 mg/kg bw for 3 weeks by gavage increased the level of kidney MDA [78]. Also, El Gendy et al. [82] reported that oral administration of 15 mg imidacloprid/kg significantly elevated hepatic LPO levels in male mice 24 h after the treatment. A significant increase in liver lipid peroxidation after administration of 1 mg imidacloprid/kg bw/day in rats exposed orally for 30 days was reported by Duzguner and Erdogan [39]. The same authors report elevated LPO in the liver of female rats exposed to a single dose of 10 μM imidacloprid intravenously [38]. Kapoor et al. [83] observed that a dose of 20 mg imidacloprid/kg/day significantly increased LPO levels in liver and kidney of female rats after oral 90-day exposure. A significant increase in TBARS was reported in the kidney of male rats after 24 h exposure to imidacloprid at a dose of 170 mg/kg intraperitoneally [42]. Oral treatment with imidacloprid at a 38 mg/kg dose for 20 and 30 days significantly increased MDA levels in the liver of rats [70]. Also, lipid peroxidation was significantly elevated in the rat’s liver after oral exposure to 30 mg imidacloprid/kg for 8 weeks [74]. In our investigation, the obtained results for lipid peroxidation, expressed as TBARS levels, revealed that no significantly higher amounts of lipid peroxidation that could damage lipids in the liver and kidney tissue of rats, exposed to low doses of imidacloprid, were produced. These results are consistent with results regarding ROS levels that decreased in the liver, significant only at the dose of 0.8 mg imidacloprid/kg bw/day, and slightly decreased in the kidney of treated rats. Different results from the investigations mentioned above could be explained by the much lower doses of imidacloprid used in our investigation.

In the present study, levels of GSH were not significantly changed either. They were similar to the control values, particularly in kidney tissue, which we can explain again with the very-low doses of imidacloprid that did not elevate ROS production. GSH, as the most abundant intracellular non-protein thiol in an organism, has a key role in cell protection against free radical damage and detoxification of various xenobiotics. It also acts as a substrate or cosubstrate for many antioxidant enzymes like glutathione peroxidase (GSHPx), glutathione reductase (GR), and glutathione S-transferase (GST) [84]. When oxidative stress occurs, GSH is consumed by these enzymes to detoxify peroxides produced by lipid peroxidation. A significant decrease in GSH levels has been shown after long-term exposure to pesticides in previous studies, including studies of imidacloprid exposure in rodents. Markedly lower GSH levels were observed in the liver of both male and female mice, while no changes were observed in the kidney after oral exposure to 15 mg imidacloprid/kg bw for 21 days [65]. The same results of decreased GSH content in the liver without change in the kidney of female rats after oral administration of imidacloprid at a dose of 20 mg/kg/day for 90 days were reported by Kapoor et al. [83]. Decreased liver GSH levels after 12 h exposure were observed in male rats exposed intraperitoneally to 170 mg imidacloprid/kg, while in the kidney of male rats, GSH levels increased after 24 h [42]. Significant reduction in GSH concentration in the kidney and liver tissue of male rats on days 14 and 28 after oral exposure to imidacloprid at a dose of 80 mg/kg bw for 28 days was found by the same group of authors [41,77]. In the study by Lafi et al. [78], 3-week exposure to 64 mg imidacloprid/kg bw by gavage significantly decreased GSH levels in the rat kidney. Significantly reduced GSH concentration was also observed in the liver of female rats exposed to imidacloprid orally at a 38 mg/kg/day dose for 30 days [70], in the liver of male mice 24 h after oral exposure to a single dose of 15 mg imidacloprid/kg [82], and in the liver of female rats treated with a single intravenous dose of 10 μM imidacloprid [38].

Besides non-enzymatic antioxidant defenses like GSH, also primary antioxidant enzymes like GSHPx, CAT, and SOD exist and they can be affected by pesticide exposure [85]. These enzymes have an important role in the protection against deleterious effects of lipid peroxidation, and their attempts to remove continuously generated free radicals firstly increase, but due to enzyme depletion that happens later oxidative damage occurs [38]. Existing studies report that imidacloprid exposure can disturb the activities of antioxidant enzymes at different toxicities, including in liver and kidney toxicity. Some studies report an increase in the activity of antioxidant enzymes, while some others report inhibition in antioxidant enzyme activity. Increased activities of antioxidant enzymes GSH-Px, CAT, and SOD have been observed in the liver of male mice 24 h after they were exposed orally to a single dose of 15 mg imidacloprid/kg [82]. In a study by Kapoor et al. [83], reduced activities of antioxidant enzymes CAT, SOD, and GSH-Px were observed in the liver tissue, but not in the kidney tissue, of female rats exposed to 20 mg imidacloprid/kg/day for 90 days. The same results of decreased liver CAT, SOD, and GSH-Px activities were found in the study by Qumsani [74] after exposure of male rats to 30 mg imidacloprid/kg in drinking water for 8 weeks. Also, 3-week exposure to imidacloprid by gavage at a dose of 64 mg/kg bw reduced CAT, SOD, and GSH-Px activities in the kidney tissue of rats [78]. Oral exposure to imidacloprid at a dose of 38 mg/kg/day for 20 and 30 days diminished SOD and GSH-Px enzyme activity in the liver of female rats [70]. The authors explained the decrease in SOD activity as a possible result of the consumption of this enzyme in the catalyzed reaction of the superoxide anion (O^2−^) into hydrogen peroxide (H_2_O_2_). Decreased GSH-Px activity in the liver tissue might be the consequence of oxidative inactivation of the enzyme protein due to accumulation of imidacloprid in the liver since GSH-Px is localized mainly in mitochondria and cytosol of the liver, which can be considered as sources of this enzyme. Acute exposure to 10 μM imidacloprid intravenously decreased GSH-Px and increased SOD activity, without changes in CAT activity in the liver of female rats [38]. The authors suggested that since no change in CAT activity was found, after insecticide exposure, firstly SOD then GSH-Px are activated. In a study from the same authors, chronic oral exposure of female rats to 1 mg imidacloprid/kg bw/day for 30 days did not produce changes in CAT, SOD, and GSH-Px activities in the liver tissue [39]. After oral exposure to a dose of 15 mg imidacloprid/kg for 21 days, no significant changes in CAT activity but decreased SOD activity was noticed in the liver of male and female mice and kidney of male mice [65]. Reduced activities of antioxidant enzymes were explained by consumption of these enzymes due to increased oxidative stress in the liver and kidney tissues. In the study by Yardimci et al. [42], the authors found decreased CAT and increased GSH-Px activity in the liver of male rats after 24 h of exposure to 170 mg imidacloprid/kg intraperitoneally, without changes in kidney tissue. The results of the study by Omar et al. [73] revealed that exposure to 14, 28, and 54 mg imidacloprid/kg bw/day for 28 days caused oxidative damage in the liver of male rats that was evident from the decreased activities of CAT, GSH-Px, and GST enzymes. In our investigation, low-level imidacloprid exposure disturbed the activities of antioxidant enzymes CAT and SOD in both liver and kidney tissues, but not significantly. Activity of GSH-Px was significantly decreased in the liver of rats exposed to the highest dose of 2.25 mg imidacloprid/kg bw/per day. This is the only result that confirms possible antioxidant capacity induced by low-level imidacloprid exposure. Such results may suggest that low-level imidacloprid exposure did not produce high amounts of reactive oxygen species like superoxide anion that could be catalyzed by SOD to a less-toxic substance, hydrogen peroxide, which could be converted by CAT and GSH-Px to water.

Besides lipid peroxidation and protein degradation, the accumulation of free radicals may also lead to DNA damage, which altogether may finally injure various important tissues, including the liver and kidney. In our study, DNA damage induced by imidacloprid exposure was detected by the alkaline version of the comet assay. This method detects a broad spectrum and low levels of DNA damage [86]. Previous studies have shown the genotoxic potential of neonicotinoids, including imidacloprid, in different target and non-target organisms [87,88,89,90], as described in detail in our previous paper [37]. Research on imidacloprid’s genotoxic potential in vital organs such as the liver and kidney of rodents is scarce so we considered it would be important to perform this kind of investigation in order to clarify its genotoxic action. Based on the results obtained, we can say that the genotoxic damage determined after exposure to the tested doses of imidacloprid in both the liver and kidney of the exposed rats was low. Considering results regarding oxidative stress, DNA instability was not driven by those phenomena. If we consider that the levels of about 5% DNA in the comet tail can still be considered within acceptable control limits [86,91], then the values we determined in the liver and kidney of treated rats are not worrying. However, we must emphasize that in order to understand and interpret the comet assay method, all measured values for a given treatment or group in the experiment should be considered. The method itself, as its name (i.e., single-cell gel electrophoresis) suggests, is based on the analysis of the levels of damage in individual cells. It is evident from these collective data that the imidacloprid-exposed group had a more pronounced response in terms of an increase in the number of cells with higher maximum values for both comet assay descriptors. This means that, unlike the negative and solvent controls, a part of the cells measured in the groups that received imidacloprid responded with increased levels of damage, which indicates impairments in the genome integrity of both types of liver and kidney cells. Despite the low group mean/median values, it should also be noted that we observed greater overall damage in rats that received the lowest tested daily dose of imidacloprid for 28 days. Their values were higher than those measured in rats exposed to the two higher doses. Possible reasons for such a response lie in the fact that repair mechanisms are activated differently over the 28 days. Exposure to higher doses of stressors (at two higher tested imidacloprid doses) may have led to an adaptation of the repair systems in rats that, due to more efficient mechanisms of repair, finally had lower levels of primary DNA damage. The same phenomenon was observed in some comet assay studies in vivo with corresponding durations of exposure [92,93]. We must also consider that during the 28-day treatment, a series of changes occur in organs and tissues at the cellular level, in which some cells with a higher level of damage die through the processes of apoptosis and necrosis. Therefore, there is a possibility that after treatment with two higher doses, some cells of both the liver and kidney tissues were highly damaged due to cytotoxic effects. It is generally known that the level of damage associated with cytotoxic effects causes destabilization and fragmentation of DNA, which, during the technical performance of the procedures in the comet assay, can be washed out of the gel and, thus, become inaccessible to the measurements.

Investigation conducted by Hassan et al. [71] revealed that imidacloprid exposure at doses of 45 and 90 mg/kg bw for 21 consecutive days significantly increased tail length in the liver of male rats, which was dose-dependent, indicating that DNA damage decreased by adding quercetin. Imidacloprid produced significant dose-dependent increases in DNA damage in hepatocyte cells of male rats treated with doses of 14, 28, and 54 mg imidacloprid/kg bw/day for 28 days. The reduction in tail length was observed in the groups receiving nanoemulsion containing clove oil and pomegranate extract [73]. Different results between our study and the above-mentioned studies could be explained by the low doses of imidacloprid that did not cause significant oxidative damage. Effects on liver DNA damage were observed in a few studies investigating the impact of other neonicotinoid pesticides. For example, exposure to dinotefuran, the latest discovered neonicotinoid, orally at a low dose of 0.001 mg/g bw for 90 days confirmed liver DNA damage with increased tail length, accompanied by affected histopathology and oxidative stress parameters, which all improved but not significantly after vitamin E supplementation in rats [94]. Also, oral exposure to thiamethoxan, a second-generation neonicotinoid insecticide, at a dose of 78 mg/kg bw for 14 days, induced increases in comet assay parameters like tail percentage, tail length, tail DNA percentage, and tail moment in liver samples of male rats [95].

Here, we will briefly address the limitations and strengths of our research. We are aware that one of the main limitations of this study is that the experimental design employed did not enable histopathological analysis, which could eventually give more precise answers to some questions about the tissue-specific adverse effects of the imidacloprid treatment. They were not conducted in parallel with all the other analyses due to restraints in the total number of animals used in the research, in order to meet the requirements of the 3R concept as much as possible. Nevertheless, we believe that the amount of novel data gathered in this study may present an important scientific contribution towards defining AOPs of imidacloprid toxicity. On the other hand, the strength of this research is that, generally, there have been no papers so far describing imidacloprid hepatotoxicity and nephrotoxicity in experimental animals by using the same set of biochemical, genotoxic, and analytical methods. Also, previous research on imidacloprid toxicity was focused on much higher doses than those we used in our study, which are expected to occur in real life and are based on recommended and safe values proposed by regulatory agencies.

## 5. Conclusions

The current study has shown that sub-chronic exposure to low doses of imidacloprid, which are within the recommended toxicology relevance values, did not significantly impair liver and kidney integrity at the biochemical and cellular levels. As it seems, these organs are less susceptible to the adverse effects of imidacloprid exposure, as shown in our exposure scenario. In that view, the obtained results provide additional evidence to our previous research [37], from which we can draw the common conclusion that at low doses, the brain tissue was the most responsive to imidacloprid treatment. We found detectable levels of imidacloprid in the urine of rats, pointing to its rapid excretion rate. However, these low doses did not provoke a significant oxidative stress response, and resulted in low genotoxic effects. A significant inhibition in ChE activity, but only at a dose of 0.8 mg imidacloprid/kg bw/per day in both tissues, was noticed.

Considering the results were obtained on a rat model with the same doses of imidacloprid in this and our previous study [37], future research can continue in the direction of testing other animal models. We believe that the use of various specific and sensitive tests for neurotoxic effects could contribute to a better understanding of the adverse effects of imidacloprid.

## Figures and Tables

**Table 1:** Results of alkaline comet assay expressed as tail intensity (DNA%) and tail length (µm) measured in small-sized (non-parenchymal) and medium-sized (parenchymal) liver cells of male Wistar rats treated orally for 28 consecutive days with imidacloprid applied at three doses, and in the corresponding controls (*N* = 5 rats per group).

		Small-Sized Liver Cells (Non-Parenchymal Cells)		Medium-Sized Liver Cells (Parenchymal Cells)	
Experimental Group		Tail Intensity	Tail Length	Tail Intensity	Tail Length
Negative control (NC)	Median Min–Max	0.07 ^PC^ 0–8.88	17.08 ^PC^ 5.83–38.33	0.05 ^PC^ 0–9.80	20.42 ^PC^ 11.67–39.58
Positive control (PC)	Median Min–Max	3.29 0–18.07	22.92 12.5–44.17	1.96 0–16.32	25.42 12.5–39.17
Solvent control (SC)	Median Min–Max	0.23 ^PC^ 0–14.55	18.33 ^PC^ 10.83–45.00	0.27 ^PC^ 0–9.92	22.08 ^PC^ 9.58–44.58
0.06 mg/kg bw/per day	Median Min–Max	0.18 ^NC,PC^ 0–10.10	17.08 ^NC,PC,SC^ 10.00–42.92	0.17 ^NC,PC,SC,b,c^ 0–9.96	20.83 ^NC,PC,SC,a^ 11.25–33.75
0.8 mg/kg bw/per day	Median Min–Max	0.05 ^PC,SC,a^ 0–11.46	15.00 ^NC,PC,SC,a^ 9.58–34.17	0.03 ^PC,SC,a,b^ 0–12.58	18.33 ^NC,PC,SC,a^ 11.67–42.92
2.25 mg/kg bw/per day	Median Min–Max	0.07 ^PC,SC,a^ 0–13.74	15.00 ^NC,PC,SC,a^ 10.00–37.92	0.03 ^NC,PC,SC,a^ 0–14.54	17.92 ^NC,PC,SC,a,b^ 12.08–37.92

Statistically significant values (set at *p* < 0.05; Mann–Whitney U test) are as follows: ^NC^—vs. negative control; ^PC^—vs. positive control; ^SC^—vs. solvent control; ^a^—vs. 0.06 mg/kg bw/day; ^b^—vs. 0.8 mg/kg bw/per day; ^c^—vs. 2.25 mg/kg bw/per day. A symbol next to a value indicates a statistically significant difference (*p* < 0.05) from the experimental group represented by that symbol.

**Table 2:** Results of alkaline comet assay expressed as tail intensity (DNA%) and tail length (µm) measured in kidney cells of male Wistar rats treated orally for 28 consecutive days with imidacloprid applied at three doses, and in the corresponding controls (*N* = 5 rats per group).

Experimental Group		Tail Intensity	Tail Length
Negative control (NC)	Median Min–Max	0.04 ^PC^ 0–10.42	15.83 ^PC^ 8.75–37.5
Positive control (PC)	Median Min–Max	2.24 ^NC^ 0–31.24	22.08 ^NC^ 11.67–48.33
Solvent control (SC)	Median Min–Max	0.26 ^NC,PC^ 0–17.75	16.04 ^NC,PC^ 7.92–43.33
0.06 mg/kg bw/per day	Median Min–Max	0.16 ^NC,PC,SC^ 0–20.84	16.25 ^NC,PC,SC^ 7.08–42.5
0.8 mg/kg bw/per day	Median Min–Max	0.05 ^NC,PC,SC,a^ 0–25.13	15.00 ^NC,PC,SC,a^ 7.92–43.33
2.25 mg/kg bw/per day	Median Min–Max	0.06 ^NC,PC,SC,a^ 0–17.80	15.42 ^NC,PC,SC,a,b^ 9.58–39.17

Statistically significant values (set at *p* < 0.05; Mann–Whitney U test) are as follows: ^NC^—vs. negative control; ^PC^—vs. positive control; ^SC^—vs. solvent control; ^a^—vs. 0.06 mg/kg bw/day; ^b^—vs. 0.8 mg/kg bw/per day. A symbol next to a value indicates a statistically significant difference (*p* < 0.05) from the experimental group represented by that symbol.

## Data Availability

The data presented in this study are available upon request from the corresponding author.
